# Beyond the Normative Family Meal Promotion: A Narrative Review of Qualitative Results about Ordinary Domestic Commensality

**DOI:** 10.3390/ijerph18063186

**Published:** 2021-03-19

**Authors:** Fairley Le Moal, Maxime Michaud, Carol Anne Hartwick-Pflaum, Georgia Middleton, Isabelle Mallon, John Coveney

**Affiliations:** 1 Centre Max Weber-UMR 5283, Université Lumière-Lyon 2, 69676 Bron, France; isabelle.mallon@univ-lyon2.fr; 2 Social Sciences Group, Institut Paul Bocuse Research Center, 69130 Ecully, France; maxime.michaud@institutpaulbocuse.com; 3 College of Nursing and Health Sciences, Flinders University, Bedfork Park, SA 5042, Australia; georgia.middleton@flinders.edu.au (G.M.); john.coveney@flinders.edu.au (J.C.); 4 R&D, Mars Food Global, 3261 LW Oud-Beijerland, The Netherlands

**Keywords:** family meal, health norm, practices, interactions, conflicts, emotion work, ethnography

## Abstract

There exists a normative representation of family meals in contemporary Western societies which is promoted as imperative through public health programs, larger discourses and by some studies in the nutritional and public health research fields. Family meals, also called domestic commensality, are represented as convivial events and are associated with positive health and wellbeing outcomes but there is minimal evidence to show they are beneficial for family members and it is not known which aspect of the family meal could be responsible for these alleged benefits. This normative family meal image is based on a representation of the family as a peaceful unit exempt from external constraints. This narrative literature review of qualitative studies of family meals seeks to put forward the underlying premises of this representation and compare it with reports about actual practices. The results emphasize that eating together is still practiced and remains valued by family members, which is in contrast to discourses lamenting the decline of the family meal. However, the valorisation and recurrence of family meals depends on class, gender and cultural positions. There is a gap between the norm of healthy or convivial and achievable family meals, which can reinforce the so-called “mental load” and “emotion work” of those in charge of feeding the family and heighten inequalities within the household. In fact, there are many challenges to family meals which originate from external constraints or are inherent aspects of family life. The results from this review suggest that we should focus on family meals by taking into account the food work surrounding it and focussing on the interactional aspects of family meals. Ethnographic methods allow the researcher to observe the diversities and complexities of commensality as well as family dynamics and, in doing so, could provide more realistic representations of eating within the family.

## 1. Introduction

The subject of family meals is a recurrent theme in debates about food and family life in contemporary Western societies. The family meal refers to a diversity of forms and functions but, in a broad, common sense, it implies all of the family members of a household that are present coming together at the same time and location to eat, whether the food is the same or different [1]. Family meals are also mentioned in academia by the expression domestic commensality [2,3]. The etymological origin of commensality means sharing the table or the food [4]. It comes from the Latin “cum”, meaning with and “mensa”, the table or the food, but in practice, it is not restricted to such furniture and there are debates as to whether the same food needs to be eaten for commensality to happen [5,6]. There exists ordinary commensality, referred to as routine shared meals between close family members, and extraordinary commensality which indicates an exceptional occasion, usually with a larger commensal circle, a more elaborate or exceptional menu or a meal taken at a different location [2]. This review concerns ordinary commensality, or family meals and both expressions will be used throughout the paper.

Family meals are promoted in public health campaigns, the media and in the public opinion as a healthy and a convivial practice, by referring more or less accurately to results from research studies [7]. Yet, this type of promotion of family meals is misleading as, so far, only correlational associations have been shown between domestic commensality and positive health and wellbeing outcomes, has shown by systematic reviews and meta-analyses in this area, largely focussed on the frequency of family meals [8,9,10]. However, the research field on family meals is still lacking a social science review of papers that tackles this paradoxical phenomenon of promotion of family meals for health and wellbeing reasons despite strong scientific evidence supporting these claims.

Family food practices are a popular area of research that easily gain the attention of the larger public and the media. This is particularly true today as diseases attributed to food consumption constitute a great public health challenge, but which risk management often fall on families, in neoliberal contexts. The sociologist Grignon argues that the field of dietary expertise can be influenced by general opinion, reinforcing popular misconceptions, which can align with certain ideological and political understanding of society [11]. According to this and in line with the sociologist Poulain, we argue that it is necessary to “*demonstrate the socially constructed dimension of concepts used by sciences that ‘touch’ food*” [12]. This is all the more necessary for family meals, where “*the normative and the performative are very far apart*” as the anthropologist Wilk claims [13] (p. 428). Researchers in social sciences have already analysed and described this phenomenon as the “*family meal panacea*” [14] or “*family meal imperative*” [15]. Social scientists have also observed that the convivial [16] and happy meal [13] terminology is also often used to refer to family meals, implying that people coming together to eat necessarily generates a positive experience. This discourse is associated with a lament of declining family meals, which is what the sociologist Murcott has observed and describes as an anxiety that the family meal as a social event is disappearing and that this loss is the cause of important moral, health and social issues [17]. Such a review, that encompasses the main social sciences papers on what we broadly englobe as the “normative family meal promotion”, is necessary to move forward in the research fields of family meals.

## 2. Research Questions, Aim and Objectives

This review seeks to answer the following question: What are the conceptual and empirical limits to the normative family meal representation?

We aim to deconstruct the normative family meal representation through a sociological perspective and by contrasting it with results from qualitative studies of ordinary family meals.

This review therefore addresses the following objectives:

1. To identify the underlying premises and inherent issues of the normative family meal promotion.
2. To determine whether there is solid evidence enabling us to state that families are no longer eating together.
3. To put forward the social and gender variations in the practices and representations of family meals.
4. To identify the challenges that families face in the orchestration of and during family meals.
5. To identify the methodological gaps in the current research on family meals.

## 3. Methods

We have adopted a narrative approach for this literature review of qualitative studies and opinion papers about family meals. This was a suitable method that could provide a broad enough scope to deconstruct the normative family meal promotion and identify the following key areas of interests [18,19]: the premises and limitations of the normative family meals promotion, evidence of declining family meals, family members representations and practices of family meals and the variations according to social classes and gender status as well as potential methodological biases in the study of family meals. This type of literature overview is commonly used in social sciences [20].

The scope of this review included several types of papers published in English and in French: peer reviewed journal articles, book chapters, media articles and public health dietary guidelines. The initial search was very large: we first aimed at placing the family meals in the broad context of family food consumption. We identified the major and most recent publications in the sociology and anthropology of food [12,21,22,23,24,25,26]. This enabled us to identify previous publications on the topic of family food practices in general and family meals in particular. From then on, we also searched for databases with a large spectrum of key words that would enable us to find articles relating to the family meal as a social event (Table 1).

The following databases were used that enabled us to conduct a wide search of articles from the social sciences: Sage Journals, Jstor, Taylor and Francis Online, Semantic Scholars, Springer Link, Wiley Online Library, Science Direct, NCBI, Pubmed, Cairn, HAL and Persée. Google search engine was also used to identify media articles referring to family meals. The search process was also done by reviewing the references lists of the selected documents according to our topics of interests and research questions, thus providing further papers to review. As this is a narrative review, the searches were not systematic nor exhaustive but we navigated through these databases in an iterative process, keeping in mind our research questions, topics of interests and inclusion criteria.

This review is based on qualitative papers, but we included some quantitative studies as well to identify data on the frequency of family meals. To be included in the review, the qualitative studies had to be based on semi-guided interviews, focus groups, video and photo elicitation, in person observation or ethnography. We defined “family” as a household with at least one parent and at least one child between the ages of 0 and 18.

## 4. Results

### 4.1. Representations of the Normative Family Meal

#### 4.1.1. Public Health Promotion

In France, the family meal is advertised in the latest dietary public health program as a way of achieving a better diet and reducing the risk of obesity [27], based on meta-analyses of studies, conducted by Dallacker and colleagues and Hammons and colleagues, that associate family meals with nutritional and weight benefits [10,28]. It is notable that the studies included in these meta-analyses were most commonly conducted in the United States; few of them were conducted in Europe and none in France. The French National Nutrition and Health Programme website states the following: “*people who regularly eat meals together as a family would have a better diet than others and less risk of obesity*”, and family meals are a “*proven way to fight obesity*” [29]. Here, the meal is targeted as a medium for dietary and weight normalisation. Family members are also urged to have regular meals together as they are “convivial” occasions [29]. We can observe a similar promotion of domestic commensality in Anglo-Saxon countries such as in the United States [30], the United Kingdom [17,31] and Australia [15,32]. The nutritional public recommendations in Australia are similar, although the conviviality aspect is less present, with messages such as: “*eat with other people not TV*” [33].

#### 4.1.2. Intervention Programs

Recommendations promoting shared meals are not necessarily adopted as public health campaigns based on individual capacity of change can be ineffective [34]. However, intervention programs exist that aim at increasing the frequency of family meals within households, promoting its alleged benefits. The non-profit and non-government US online health program called The Family Dinner Project is an example of this [15,35]. In Australia, domestic commensality is also encouraged through education programs [36] or via organisations such as the Healthy Kids Association. This non-profit, non-government organization seeking to support and influence healthy food choices for children states the following on their website: “*research shows that families who eat together regularly (that’s more than three times a week) have shown to have more positive outcomes when it comes to health, family relationships and social development*” [36]. They also acknowledge that it “is next to impossible” to eat together as a family because of long working hours and children’s activities but that families should endeavour to do so anyway.

#### 4.1.3. Media Representations

There are no known interventions programs such as those presented above in France; however, the media representation of the family meal has been a lament of its decline. While previously the discourses were usually about the rise of so-called ‘individualized eating habits’ that would “threaten the very idea of being able to eat together” [37], there are more nuanced discourses about the family meal today, in some cases arguing that “the injunction to eat together is completely unfounded” [38]. However, more specialized media communication, targeted to parents and more specifically mothers, still encourage the benefits that family meals supposedly provide. Some acknowledge the difficulties people are faced with when tasked with organizing and arranging the meal, but the overarching message is that “it’s worth the effort”, because of the positive outcomes in terms of dietary health, positive communication [39], and school success families stand to gain from it [40]. In the context of the COVID-19 pandemic caused by the SARS-CoV-2 virus, there is an evolution in the media discourses about the family meal, during which lockdown episodes and work from home arrangements would constitute unique “comforting” occasions for families to eat together more often or better [41,42,43,44].

#### 4.1.4. The Strength of the Normative Model of Family Meals

Many discourses of family members point to aspirations that are similar to this representation of a family meal as a positive experience and a necessity [45]. The way the meal is discussed refers more or less directly to an idealized and normative version of family life, that has the ability to make parents feel like they are not doing things correctly if they are not done according to this ideal [46]. DeVault’s study sheds light on the work of feeding the family and the potential oppressiveness it can have for mothers. One mother in her study compared her practices with her childhood memories:

*“My mom was home. And it really makes a world of difference. She always had good meals on the table… It was more of a family thing […]. Now it’s like helter skelter routine. If we’re all home fine, if we’re not then we just work around it […]. There are a few times when I really regret it. I regret not having a family routine. It feels like, you know, your kids are being shuffled around, and you’re being shuffled around. And there are times when I get this real craving to stay home, stay home and play housewife. But then you know there is no way in hell that you could afford it. It’s a matter of economics. You have to do it in order to survive”*.

[46] (p. 48)

Bowen and colleagues remind us that the promotion of the family meal in the United States began during the industrial area, when the nuclear family was constructed as a safeguard against dangers associated to the public realm. However, usually only affluent households could achieve such a norm, generally by externalising other care and housework activities or with mothers staying at home [30].

In France, the family meal has also been constructed, during the nineteenth century, as bulwark against the dislocation of a certain type of family that would be threatened by modern life [47]. There is a representation of a contemporary pattern of eating referring to three meals a day, according to regular schedules, in “appropriate” places, usually sitting down at a particular table and with a certain number of courses at the meal. The French still refer to this strict pattern even if they do not comply with it in a rigorous manner, in particular in the structure of the meal [48]. As the sociologists Grignon and Grignon remind us, this was probably always the case, as “*the pattern of meals is an example to be followed, a kind of template, an ideal, too, to which one can approach but one can never wholly realize*” [48] (p. 253). This pattern is also commonly associated with home cooked meals and a convivial atmosphere [47]. What is indeed remarkable, even today, is that this model of regular family meals is strongly valued, even though evolutions of contemporary life make its practice difficult, if not impossible, on a daily basis [8,49]. Surprisingly enough, as Marenco notes in 1992 about the pattern of regular family meals, “*the absence of reference of this model has an exceptional character: it leaves room for no other*” [47] (p. 6).

### 4.2. The Durability of a Nostalgic Approach to Family Meals

The lament of declining domestic commensality in Western societies [17,45] implies that families do not eat together enough or they do so in improper forms, with the presence of technological devices (such as TV and phones), for example [50] (Table 2). This discourse is associated with critiques of individualised food preferences and other practices commonly attributed to contemporary life such as eating out, mothers working full time outside of the home and children’s extracurricular activities. It also suggests that some potentially positive aspects of domestic commensality, such as communication opportunities or conviviality, cannot be reproduced elsewhere throughout family life. However, as the sociologist Fischler argues, the question of whether family relationships are dissolving with the decline of the family meal is subject to ideological and moral biases:

*“The reason is probably that the deepest issues at stake are of essential social significance and carry fundamentally moral undertones. After all, the sharing of food involves the very structure of social organization, no less than the division and allocation of resources”*.

[51] (p. 529)

Additionally, there is evidence that many households still have regular family meals, contrary to general belief (Table 3). Researchers have shown that this promotion of an ideal family meal families should endeavour to achieve existed in the Edwardian period in Britain. This suggests that the promotion of a normative family meal, and fears of families not achieving it, are not new [17,31] (Table 2).

### 4.3. Searching for Health Benefits of Family Meals: A Preventive Approach

The normative representation of the family meal is linked to the belief that eating alone within the family household leads to unhealthy food behaviours [52,53] (Table 2). It represents a form of ‘gastro-anomie’, according to the sociologist Fischler [54], where food norms are destructured. This is connected with a wider lament of increased individualization processes and of the reconfiguring of food sharing norms. The stigmatisation of eating alone could be associated with the belief that commensality helps regulate food intake [51]. Moreover, eating alone is often associated with eating in front of screens, such as phones, computers, televisions and electronic tablets [55], indicative of the negative image of eating alone, as if using technological devices during meals necessarily implies isolation and is altogether detrimental to the experience of the ideal meal [50]. Having regular family meals has been associated with numerous positive health and wellbeing outcomes [8], particularly in terms of dietary and health benefits for children, including body weight [10,56].

Some studies have reported a protective relationship between family meals and adolescent risk behaviours [57] and disordered eating behaviours [58]. However, most of the research that seeks to provide evidence on the benefits of family meals is correlational, meaning a causal relationship cannot be determined. A recent systematic review of intervention studies that targeted family meal behaviours and measured family meal outcomes demonstrated that a causal relationship between family meals and health and wellbeing outcomes has yet to be proven [8]. Middleton and colleagues’ review also reported that there is a scarcity of intervention studies specifically targeting the family meal, and a lack of consistent tools to measure family meal outcomes, thus preventing a proper critical examination of the impact these interventions may have on improving or changing family meals. Additionally, there is no evidence as to which component of the family meal—the frequency, the meal environment (the general mood of the eating together occasion, who is present, if technological devices are used during the meal, for example) or the food served—would be responsible for positive health outcomes.

Positioning mundane family meals simply as a healthy practice is a rather simplistic way of addressing a phenomenon that is complex and highly dependent on social and cultural norms and discourses, and that is not achievable nor desirable for many. The association of food and health is not new, and it has garnered the attention of anthropologists for a long time. There are inherent ambivalences in human food consumption. One of these is the complexity of the relationship between health and food, because the latter is an indispensable source of energy, nutrients and health but also a potential cause for illnesses and even death [12]. What is notable with the normative family meal promotion is that it is not only the content of the meal that is supported for health reasons, but also the form of eating, in particular how often and with whom. This association is linked to the medicalisation of society, where more and more aspects of life are covered by a preventive health approach [59]. Although it may not be a medicalisation of domestic commensality in the strict sense of the term, it corresponds to a healthification process, where each practice is examined in relation to its health benefits or dangers.

The health meanings that individuals adopt are varied and intimate and may not overlap with the construction of the family meal as a healthy practice [60]. Encouraging domestic commensality through a preventive discourse implies that the association of food practices and health is evident. This disregards the possibility that eating together can have a variation of meanings for people, depending on their gender, age, employment status, social class and culture. Public health dietary guidelines can be interpreted differently according to socio-economic positions [21,26,61]. Depending on different social positions and stages of life, the link between food practices and preventive health are not necessarily adopted or perhaps even known [26,62]. The sociologists Régnier and Massulo explain that for families in France with higher socio-economic backgrounds, food is generally perceived in terms of its long-term health prevention possibilities. Conversely for those from lower socio-economic backgrounds, food is used as a curative means more temporarily, in the case of a disease such as a diagnosis of diabetes that would lead to a change in food consumption and the adoption of a new diet [63].

The way that domestic commensality is presented as a healthy practice can be associated with the way it was constructed, during the nineteenth century, as a central role in the institution of the family [47]. Families have been coming together to eat for centuries. However, the historian Marenco argues that “the novelty is that this meal taken together now eludes the category of daily practices of which nothing is to say, for which there is no model, to be explicitly assigned a central role in the domestic sphere and the functioning of the family” as a “model of manners” [47] (p. 113). Some authors argue indeed that the medicalisation of food practices is linked to the weakening of the family and religious institutions [12] and preventive health practices becoming the norm [59].

### 4.4. Domestic Commensality Is Not Conviviality

Even before family mealtimes began to be associated with measurable health and wellbeing benefits, they were simply encouraged because they are supposed to be convivial. Commensality is even said to transform the perception of the food to such point that pleasure from eating could only happen in this context [64]. However, if eating together can produce conviviality, it is not always the case nor does it necessarily happen throughout the whole meal [2]. There are few studies that question what conviviality actually means in the context of family meals [16]. It is usually thought of in rather simplistic terms as the pleasure of being together while eating. For a meal to be convivial, all the family members have to adhere to the same collective desire of friendliness, shared love, harmonious communication and equality [16]. This common association of family meals with conviviality is linked to the representation that the family is necessarily conciliatory and peaceful; however, domestic violence and the many conflicts and tensions that exist in family life are proof of the contrary (Table 2). Conviviality during a meal depends on who is present, but also on the pre-existing social and cultural conditions of the “convives” (a French term for those who share a meal together) [16]. Conviviality is not a static aspect of sociality but an ongoing process that can be intertwined with forms of tensions. As the anthropologist Heil argues, conviviality can be a fragile balance between cooperation and conflictual situations [65].

### 4.5. Ignoring the Impact of Systemic Inequalities on Family Food Practices

The family meal imperative builds on the premise that parents—and more specifically mothers—are entirely responsible for the choices they make for themselves and their children regarding health. It is expected that individuals should adapt their food consumption towards preventive practices in order to reach individual autonomy and become moral and virtuous citizens [60,66,67]. However, positioning parents as solely responsible for their children’s food practices incorrectly places the consequences of systemic inequalities on the backs of caregivers. In doing so, this representation of parenting obscures the multiple structural inequities that shape the conditions within which parents make daily choices [61,68]. These are especially inherent to gender positions, socio-economic and parental status. The sociologists Bowen, Brenton and Elliott argue that the numerous recommendations that Americans receive in terms of food, notably that of making home cooked meals, draw on “popular notions about individual responsibility and hard work that resonate with the belief that the United States is a meritocracy” [30] (p. 222). They advocate for an alternative way of thinking about family food practices:

*“Trying to solve the environmental and social ills of our food system by demanding that we return to our kitchen en masse is unrealistic. At best, it is a weight of responsibility that will most likely be felt by women who tend to occupy this space already. We need to change the way we think about food, family meals and inequality”*.

[30] (p. 223)

A study of the sociologist DeVault of family food practices of a diverse group of American households has previously highlighted the way families struggle and try to make do with the impact of enduring social problems on home dynamics: “individuals find solutions to these problems of everyday life—some relatively easily and some at great cost. But individual adjustments do not solve enduring social problems” [46] (p. 3). As the anthropologist Wilk reminds us in his argument about the idealisation of the ‘happy family meal’, this individualisation of social problems constitutes a paradigm inherent to neoliberal policies and “*renders the failure of policy and law invisible and denies the importance of inequality and social discrimination. It turns legitimate social problems into personal moral issues, which are addressed through exhortation and preaching, often glossed as ‘education’*” [13] (p. 413). Such a paradigm also ignores the influence of the food industry on consumption practices and food preferences of families.

### 4.6. Families Still Eat Together

There is evidence that many families across Western societies are still eating together (Table 3). In France, for example, families still mainly eat together in the evening, with six or eight people out of ten having dinner with their family [69,70,71]. The dinner is the meal in France that family members share the most often. The normative pattern is that it usually happens at home on weekdays, at about 7 PM (but later in Paris) and lasts about 40 min [48]. There is also evidence that families in Australia are still having regular meals together with six adolescents out of ten declaring their previous dinner was a family meal [5]. Surveys about the frequency of family meals in Nordic countries indicate similar results, varying between five to six adults out of ten declaring having a family meal in the past twenty-four hours [72]. In the US, 53% of families declare eating together seven or more times per week [73], while in the UK, that rate is down to 51% of respondents eating together on a daily basis [70]. Of course, this evidence needs to be compared with previous results to define an evolution but, considering the many barriers to having regular family meals (Table 4), we can still argue with confidence that discourses regretting the disappearance of domestic commensality are often at odds with the reality. However, most of these results are not that recent and it is difficult to compare between them as there are sometimes great variations in the methods used (Table 3). There is also rarely a clear and common definition of what family meals are. This may be because forms of domestic commensality vary and therefore make the analysis of its frequency more difficult to grasp and also because of the transformations to the rhythm of work and rest. It is often the frequency of eating occasions that are taken into account and the duration of meals, but there is a lack of recent evidence about the family meal environment. Indeed, a high level of synchronisation of mealtimes (family members eating at the same time) within households does not mean that family members are actually eating together at the same place.

### 4.7. Social Variations in the Practices of Family Meals

Just as food preferences and habits are socially constructed, the practices of domestic commensality vary according to the family members’ social positions (Table 4). While previous research has explored the social diversity of food consumption, in particular food preferences [26,61,74], there are fewer studies that investigate the social differentiation in the ways of eating. DeVault has described some differences between middle- and working-class families in their relationship to eating together, in her study of a diverse group of American households [46].

Middle class women who are working professionally outside of home put effort into the conversations with children during the meal, while discussion was more an issue of contention between husbands and wives for some working-class households [46,75]. DeVault also reported that some working-class women shared the middle-class notion that meals should be occasions for family communications, but that these aspirations usually resulted in conflict during meals, as they would not necessarily correspond to the partner’s expectations of family meals [46]. There also exists social differentiation in the organisation of meals in families in France: while higher-class families value that all the family members eat the same food during the meals, leaving less room for negotiations with children, while children from lower classes have more agency in the choice of the food they eat [62,76].

### 4.8. Challenges of Family Meals

#### 4.8.1. Barriers to Having Regular Family Dinners

Many households face barriers in the daily orchestration of family meals, such as scheduling conflicts and lack of time, limited resources, scarcity of help, tiredness, lack of skills or confidence [8,30,77,78,79,80,81] (Table 4). The existence of these barriers is what differentiates everyday domestic commensality from exceptional commensality. Even for families who regularly manage to eat together, they can still face challenges once the food is on the table and the family members gathered.

#### 4.8.2. Challenges during Family Mealtimes

The debate about family meals should not concern only their recurrence, the quality of the meal environment needs to be taken into consideration as well (Table 4). Shared meals are often the site of difficulties experienced by family members and can be unpleasant occasions [8,13]. Conflict can arise from the food served at the meal, as a result of difference in taste preferences, eating disorders or disordered eating behaviours, or from children confronting parental authority through food refusal and resistance of mealtime rules [8,30,82,83]. Conflict can result from children’s disruptive behaviour at the meal, such as being messy, distracted, not sitting “properly” and fighting with siblings [8]. Grieshaber’s 1997 Australian ethnographic study of family mealtimes found that children’s resistance and negotiation of parental authority and rules were “*integral parts of daily interaction and practice*” [83] (p. 664). Expression of family hierarchies, pressure and control over children are also common aspects of family meals [13,15,16,45,84]. It exacerbates power relations at the heart of the domestic space between children, women, and men [49]. The family meal can be loaded with so many expectations, argue Bowen and colleagues, that “*the more the family meal becomes a symbol of good parenting and proper family life, the more dinner feels like a pressure cooker*” [30] (p. 75).

#### 4.8.3. Gendered Aspects of Family Meals

An exploration into family meals must inevitably include a discussion about gender inequalities. Women have been reported to continue to do the majority of food work in many countries, such as in France [21], Australia [85], Nordic countries [86,87], Canada [67,88] and in the United States [46,89]. The disproportionate division of work is partly based on implicit gender norms that structure family life. Women are expected to maintain the health and wellbeing of family members as part of the accomplishment of motherhood, as a means of developing moral identities as good mothers [84,87,88,90]. They are also expected to carry the mental load of food work and implement healthy diets as an expression of femininity, which has been observed in Australia as well as in France [21,90]. Mothers’ role about food at home is also often connected to family cohesion and conviviality [16].

However, mothers who carry the bulk of the family food work tend to experience feelings of guilt and anxiety, because the ideals of healthy eating connected with good mothering are difficult to achieve [16,91]. This results in food work having the potential to be oppressive for women. These feelings are stronger for mothers situated in middle and higher classes than for mothers of lower classes, since the latter face other imperatives than those of preventive health practices, such as providing enough food for the children [61]. However, it should be noted that these are counterbalanced by a sense of reward when the ideals are achieved [46]. Studies have reported that fathers can also feel rushed and stressed when having to cope with food work, but they very rarely express guilt and anxiety as mothers do [91]. This discrepancy in the experience of family food work is linked to a normative dimension of mothers’ identity, which is not the case for fathers.

Urging families to come together regularly to eat may only reinforce gender inequalities already experienced by mothers [7,30]. Not only does the normative family meal promotion need to be analysed in light of gender inequalities, we also need to take into account social variations in the relationship to shared family meals. The normative aspiration of family meals as a convivial event, with harmonious family communication falls on mothers as well [7,16] and being able to reach these aspirations when we know there exist many challenges before and during mealtimes can be emotionally challenging as well (Table 4).

#### 4.8.4. Food Work, Emotion Work and Family Meals

Orchestrating a family meal is part of the daily so-called “food work” of feeding the family as DeVault observed. Restrictive definitions of “food work” include the tasks of meal planning, shopping and meal preparation [92].

*“Most people do not think of themselves as working when they sit down to eat with the family. Often (though not always), they are enjoying eating themselves, and enjoying the companionship of the others in their households […]. But the difficulties that may arise, especially for parents who have other work as well, provide occasions when the efforts required at mealtimes become visible”*.

[46] (p. 51)

More inclusive approaches cover the eating occasion itself and include the mental load [93] of being in charge as well as the “emotion work” [94] that feeding the family can imply. Fielding-Singh considers it the “*invisible work of thinking what everyone will eat*” [95] (p. 99) while Wright and colleagues define it as the “*emotional and domestic management of children’s eating*” [74] (p. 422). Family meals are rooted in a contradictory framework of pleasure and struggle. This means there may be some key aspects of domestic commensality that could be understood through the notion of emotion work. DeVault [46] and Hochschild [96] have begun discussing, in the 1990s, the importance of emotion work in the activities of feeding the family. Emotion work is the private manifestation of emotional labour, which Hochschild refers to as the efforts required to “*induce or suppress feeling in order to sustain outward countenance that produces the proper state of mind in others*” [94] (p. 7). Since then, food work has been more directly approached as emotion work [97] but it is usually the food provisioning and preparation that are associated with feeling efforts. There is still a dearth of studies that examine this aspect.

### 4.9. Methodological and Conceptual Limits in the Study of Family Meals

The results from the literature review have also pointed to some methodological and conceptual limitations in the current approach of family meals.

#### 4.9.1. Including Fathers

The differences in food work practices between mothers and fathers go beyond disparities in time use. The literature available on fathers reports that they are less committed to healthy eating and are more interested in choosing food for pleasure [95,98], to the extent that some mothers report preferring to do the food work themselves, rather than letting fathers do it [88,95]. This is to be put in context again with the fact that fathers are less subject to normative framing when it comes to food. Some studies suggest that men are more committed to getting children to eat rather than getting them to eat well: they would put more pressure on children to eat quantities of food [99] and do not restrict foods in line with health beliefs as much as mothers do [100]. It seems that fathers tend to favour the principle of pleasure and sociability when considering food choices, for themselves and their children rather than health and care, primarily considered by women [98]. In Australia, in particular, fathers have been reported to favour the family meal as an opportunity to connect and communicate with children and place less importance on children’s eating behaviours [101,102]. The findings on fathers’ experience of family meals remain incomplete. Their positions regarding domestic food work are sometimes only [85] or partially discussed through mothers’ discourses [95,98]. This lacuna is often explained by the fact that mothers are generally the main person responsible for food work, and by the absence of fathers responding to call for research participants [98], but it nevertheless remains problematic since they still influence food practices [50]. While there are recent studies that have since attempted to address this gap by including father’s [98,103,104], some offer a limited understanding of family food practices through interviews with fathers only, ignoring the interactional aspect of family food activities and their female counterpart’s experiences [31,102,103,105,106,107]. This bias robs us of a balanced understanding of domestic life. Additionally, interviewing fathers alone may not provide sufficient evidence of their role in family life. Anette Lareau explains the inadequacy of interviewing fathers to gather this understanding and argues that in person observations are more suited to “*capture the fluid and fleeting exchanges in the routines of daily life*” [108], of which meals are a part:

*“In our own case, it was repeated field observations inside families that brought to our attention the many positive contributions fathers make. Without the observational part of our study, we might have added to the number of studies portraying fathers as deficient in key areas of family life”*.

[108]

Although fathers are generally reported to be less involved than mothers in domestic tasks, they still have a significant influence on children’s health and weight status [106], food preferences and practices [61,107] and food decisions for the whole family [108], although these results provide only correlational associations [109]. Moreover, the tendency to study family food practices exclusively through mothers’ experiences might undermine fathers’ progress in their appropriation of domestic practices and may serve to reinforce the tendency of positioning mothers as the main person responsible for feeding the family [110].

#### 4.9.2. Including Children

Family food practices, including the family meal, can also be a negotiation with children [76], who often contest and resist adult rules [83,111]. They influence family food purchases [112], and their preferences impact the entire family’s diet [113,114] and can constitute barriers to the implementation of parents’ ideals of a healthy diet [74]. Children should be taken into consideration when studying family food practices because of the impact children can have on food choices [46,84,87]. A process of reverse socialization can take place, where children provide food knowledge to parents and other family members [97,115]. Maurice has shown, however, how the influence of children on family food practices is also socially situated. Children from middle and high classes have less agency on family food practices and choices than children from lower classes [76]. Whether or not they influence the food choices, children influence eating occasions through their own behaviours and practices during meals, which can cause conflict with their parents.

Reports from children’s experiences of domestic commensality can be quite nuanced as well. While family meals are generally discussed by children in positive terms [116], some studies mention that they can also be negatively experienced, although this aspect is rarely emphasized [117]. A survey of adolescents in Australia found that 45% considered family meals to be unimportant [5]. A qualitative study in the US reports that children do not always view the mealtime rules and interactions positively, reporting disinterest in family meal conversations, and disliking being forced to eat the food served [118]. However, to our knowledge, there are very few other qualitative studies that examine children’s experiences of family meals.

#### 4.9.3. Focusing on Family Interactions and Relationships

Not only does each family member take part in the interactional process of family food practices but the family must also be approached as a group since, as Lareau states, the “*whole is more than the sum of its parts, […] with members interacting in a fluid and dynamic fashion*” [108] (p. 429). The sociologist continues:

*“Highlighting the nature of social connections in family life, recognizing them as fluid and ever-changing, is crucial to a more elaborate notion of the elements of family life. Analyses of families must necessarily, then, incorporate the different vantage points and experiences of various members of the group. Such analyses also must be attuned to interactional processes, embedded in a broader context, rather than discrete actions studied in isolation”*.

[108] (p. 429)

Cappellini and Parsons also report about the collective and interactional aspect of family meals, wherein each family member takes part in the process of making meals happen, even if there is one person who is most responsible for the food work. They argue:

*“Sharing a meal, which makes everyone ‘happy’, is not simply the responsibility of the cook (often mother) as s/he tries to accommodate the different tastes of family members. Rather it is more than the sum of the parts, it is a collective manifestation of being a family wherein each member of the family has to take part playing a specific role, or ‘doing their bit’”*.

[119] (p. 16)

If it seems necessary to be aware of this interactional process when studying family meals, there are also benefits to taking into account an even broader context, that of family relationships in general. Sociologists have warned against the biases of evaluating the impact of domestic commensality while separating it from family dynamics. A longitudinal study from the sociologists Musick and Meier testing the association of the wellbeing of adolescent’s with family dinners, showed how some of the potential benefits of family meals (in this case reducing depression symptoms and delinquency among adolescents) are the results of stronger family relationships [120]. Bowen and colleagues summarize these results as such: “*the ability to manage regular family meals may, in other words, be a proxy for other dimensions of the family environment, like strong family relationships*” [30] (p.256). Some of the benefits associated to family meals may be due to the level of family functioning.

#### 4.9.4. Food Work and Family Meals throughout the Week

In recent years, researchers have begun to study the family meal in closer detail, generally seeking to identify which characteristics of the meal are most beneficial [121]. While this closer focus is necessary, evidence suggests that we should not study eating occasions in isolation from one another. This implies that, while it is important to look further than just the frequency of family meals, even by extending the exploration to the meal environment, there are benefits to investigating several meals throughout the week, and focusing on the food work leading up to and after the meal. An ethnographic study by the researchers Cappellini and Parsons reveals there exists different types of commensal occasions throughout the week within the same family: there are ordinary weekday meals and extraordinary meals for Sunday meals and family celebrations. The authors observe “*wide discordance between expectations and ideals of family meals and lived experience of family meal*” [119] (p. 116) for ordinary family meals, while there is less discordance in the case of extraordinary meals.

Cappellini and Parsons have also observed meals from the planning to the cleaning up. This approach enabled them to make a direct connection between the food work surrounding the meal (planning, meal preparation and cleaning up) and the way the family meal actually happens. They conclude:

*“Findings reveal a link between the effort, money and time invested in making a dinner and the effort and time spent in sharing a meal. In fact, a thrifty dish becomes a thrifty meal wherein food is displayed, served and eaten in a thrifty way, saving time and effort for all the family members”*.

[119] (p. 117)

While the resources mobilized for meals during the week are limited (money, time and effort), for the extraordinary meals, which are still part of the weekly routine, the investment of resources to make them happen are significantly more important. Additionally, the family mealtime itself is quite different, especially regarding the discussion that happens at the meal, as Cappellini and Parsons report:

*“Margaret [middle-class mother] observes that given the effort she has spent on the meal, her children are called to reciprocate by doing their part, in this case talking together during the meal. Having spent more resources preparing a richer meal, Margaret expects a richer thanks in return. Her children are expected to celebrate the special gift that Margaret donates to and shares with her family. In return for such a special gift, Margaret’s sons have to share not simply richer food, rather they have to reciprocate with a specific performance (sitting down and talking)”*.

[119] (p. 122)

Looking at reasons why families will not eat together also suggests that the meals should not be studied in isolation from food work nor without at least a minimal understanding of the family’s daily life. A father in DeVault’s study explains the following, after recognizing that they hardly ever have family meals:

*“It doesn’t make any difference. Well, it does. But you’re so damn tired. It’s not the time, because you could do it if you wanted to. It just gets to where you’re so damn tired, and fed up with the way the money situation is, and you just say, the hell with it”*.

[46] (p. 53)

This shows how organizing family meals requires efforts before, during and after the meal.

## 5. Discussion

### 5.1. Discussion of Key Results

The aim of this review was to deconstruct the normative family meal representation and compare it with ordinary experiences and performances of family meals, as reported by qualitative studies. An apparently simple discourse about the family meal, which only portrays it as being a healthy practice and a convivial moment, disregards many of the actual challenges and the variety of practices of family meals. Not only have we yet to prove a correlational relationship between family meals and health benefits, but this association of domestic commensality with health benefits needs to be considered as well in the broader context of the healthification of food practices. If such a preventive health approach is supported, it does not necessarily correlate with the diverse relationships of food practices and health adopted throughout social classes and gender status. In fact, although there is evidence that families still eat together, showing a strong attachment to the norm, this type of promotion of family meals is not new; it already existed at the end of the nineteenth century, in countries such as the US or France, and it is based on a particular ideological representation of family life. It supposes that families are necessarily non-hierarchical and non-conflictual units and considers the possibilities of action of family members in isolation from potential structural inequalities and constraints. The observation of social variations in the forms and functions of family meals indicates that the normative family meal representation seems to coincide more with the practices of families from middle and higher classes than with those of less privileged backgrounds. A preventive health approach to family meals and the association of commensality with conviviality becomes a form of social and cultural distinction for some that may not be desirable nor applicable for others. Moreover, while the normative family meal promotion is formulated in gender neutral terms, women are often expected to carry out the ideals of family meals as convivial and harmonious family events, as these correlate with normative aspects of motherhood. In fact, many of the challenges of family meals, whether they are linked to the food work surrounding meals or they happen during the meal can be linked to external constraints—time, stress, conflicting schedules and priorities, lack of resources, lack of energy to cope with the expectations of family meals—or simply relate to ordinary aspects of family life with its inherent pleasurable aspects and daily struggles.

### 5.2. Main Limits of the Current Research

We have identified some limits in the current approach of family meals. Many of the studies are based on interviews or focus groups with mothers. While representing mothers’ voices is crucial as they are often the main person responsible of food work, trying to understand a family event through one single perspective has its limits. More and more efforts are put into the recruitment of fathers and children as well, but they are often interviewed in isolation from the other family members as well. While interviews constitute an appropriate method to grasp family members’ representations and experiences of family meals, they are not sufficient to understand the relationships and interactions happening during mealtimes. Moreover, interviewing fathers about family life has been proven to be ineffective, compared to observation methods [108].

## 6. Conclusions

Behind a normative representation of the family meal as a peaceful and convivial event lies a simplistic interpretation of family life where family members’ choices and possibilities of action are extrapolated out of their social, economic and cultural contexts. This normative representation of family meals is promoted in public health dietary programs, interventions programs, the media and it also aligns with many family members’ aspirations about family food practices. However, there are some discrepancies and tensions between the aspirations of family meals as convivial and pleasurable moments, where care, love and intimacy are expressed, and the actual practices that take place. Not only are there many barriers that families face when trying to share meals regularly, family meals themselves can be challenging events. In fact, the meanings attributed to family meals are socially situated, particularly in terms of the importance of family communication and the negotiation possibilities of children. In addition, the role of family members during meals seems to be quite dependent on gender positions, although this needs further investigation. Mothers, in particular, are often associated with the potential convivial aspect of family meals.

### 6.1. Limitations of This Review

This literature review has limitations. Firstly, the studies reviewed were limited to Western countries. This limits the generalisability of the discussion to non-Western countries, and our ability to compare and contrast experiences cross-culturally. This would be useful to put forward cultural variations in the role and representations of family meals. Moreover, as with all narrative reviews, which do not seek exhaustiveness, some major qualitative studies of family meals may have been missed, particularly because the search was limited to English and French papers.

### 6.2. Directions for Future Research

Our understanding of domestic commensality could benefit from more studies that go beyond discourses—that are highly subject to normative biases—and look at the practices of meals, along with the food work surrounding them and the family environment in which they happen. Our current understanding of the family meal could benefit from a perspective that does not separate it from other eating occasions—as the meanings and forms of domestic commensality often vary throughout the week—and the rest of the family food practices. In fact, what happens in terms of family relationships and interactions during family meals still seems to constitute a black box for researchers. While family meals are promoted as helping to strengthen family relationships, it is still unclear if and how they do so.

Ethnography seems to be suited to address some of these challenges identified above and grasp the complexity, richness and changing dimensions of domestic commensality. Ethnography is a grounded approach with a focus on understanding social and cultural practices and representations from the point of view of the actors [122]. It is based on in-person fieldwork, during which the researcher is in close, long term and repeated contact with the participants, otherwise known as participant observation. This immersion in the milieu is usually complemented with interviews and strictly observation methods, and sometimes the collection of objects (which could be, for family food practices, shopping lists, grocery receipts, recipe books, meal plans, etc.). Adopting an ethnographic approach for the study of family meals implies that the researcher be present during the families’ food activities, from the food provisioning and preparation, through the consumption process and cleaning up activities. This means including all of the family members in the study and taking into account all of the points of view. An ethnographic approach enables the observations of interactions and family relationships that shape and are shaped by domestic commensality. Providing results of ordinary family meals that show potential differences between the normative aspirations and the actual practices, without labelling struggles as failure may be a healthier approach to family meals, that would enable us to constitute a more inclusive and representative image of family eating practices and provide adapted recommendations for families.

## Figures and Tables

**Table 1:** Keywords used for the databases searches.

	Databases Search Keywords
English	“family meal”, “family mealtime”, “family dinner”, “shared meal”, “commensality”, “domestic commensality”, “eat together”, “eating together”, “family food practices”, “family food work”
French	“repas de famille”, “repas en famille”, “commensalité”, “commensalité domestique”, “commensalité familiale”, “ manger ensemble”, “ manger en famille », “ pratiques alimentaires familiales”

**Table 2:** The normative family meal promotion: assumption, premises and limitations.

Premise	Families Do Not Eat Together Enough or Properly	Family Meals Provide Health Benefits	Family Meals Are Always Convivial
Associations	Critique of the individualisation of food practices Critique of the introduction of technological devices during domestic commensality	Critique of eating alone (supposedly unhealthy, socially stigmatized)	Confusion between commensality and conviviality
Origins of these premises and associations	This lament is not new: it already existed at the end of the 19th century (France) and at the beginning of the 20th (UK) Fear of the dismantlement of the family	Healthification process of food practices (preventive approach) Parents are solely responsible for their health and that of their children	Representation of the family as a peaceful and non-hierarchical unit
Issues identified	Families are still eating together Perhaps families did not used to eat together before as much as imagined	There is limited evidence that family meals provide health and wellbeing benefits The preventive health approach to food is socially situated Such paradigm ignores structural inequalities	Conflicts are inherent to families The family does not pre-exist in itself, it is constructed and maintained through practices such as shared meals

**Table 3:** Prevalence of family meals: selection of results.

Authors	Year	Country (City)	Method	Sample	Results and Limits
Michaud et al. [69]	2004	France	Phone survey (+/− 30 min) 24 h recall of food consumption Monday to Sunday	3153 12 to 75 years old 1 person per household Representative sample	86.2% of respondents who live with family members “have dinner with the family” ➢ No definition of “have dinner with the family” ➢ What proportion of respondents live with family?
Pettinger et al. [70]	2006	France (Montpellier)	Self-administered questionnaires	766 64%≥ 36 years old 40%, education ≥ 3 years 5.3% unemployed 13% retired 12% students	64.5% “eat together as a household on a daily basis” Eat together as a household daily (age): 18–35 year-old: 59%; 36–50: 66%; 51-65: 71% ➢ No definition of “eat together as a household” ➢ Family composition of respondents?
Riou et al. [71]	2015	France (Paris)	Face to face questionnaires during home visits	2994 Representative sample	23% of sample: 3 meals (89%), mostly at home (89%), with the family (61.7% share meal with the family more than 75% of the time). Pattern associated with a higher income, a nuclear family (couples with or without children) and an almost non-existent sense of loneliness.
Gallegos et al. [5]	2010	Australia (Perth)	Online and paper-based survey (+/− 15 min) Part of school curriculum 24 h recall	625 15 year old adolescents 77% dual headed household Representative sample	61% indicated the previous night’s meal was “eaten at the same time and place as everyone else in the family”. Other definitions of family meals: “meal was cooked at home”, “meal included meat and vegetables”, “television was off” ➢ Day of survey?
Pettinger et al. [70]	2006	England (Nottingham)	Self-administered questionnaires	826 72% ≥ 35 years old 26% ≥ 3 years education 4% unemployed 10% retired 3% students	51% reported eating together as a household on a daily basis 18–35 year-old: 47%; 36-50: 46%; 51–65: 71% ➢ No definition of “eat together as a household” ➢ Family composition of respondents?
Kjærnes (ed.) [72]	2001	Nordic countries	Phone survey 24 h recall of eating events (+/− 15 min) Monday to Sunday	Representative samples(≥15 years old) Denmark: 1202 Finland: 1200 Norway: 1177 Sweden: 1244	Households: couple with child(ren) Family meal: meal eaten at home with the entire household, the food eaten is hot Denmark: 66%; Finland: 51%; Norway: 60%; Sweden: 57% ➢ Restrictive definition of the family meal
Sobal and Hanson [73]	2011	US	Phone survey “In a typical week, how often do you eat a meal together with the family members who currently live with you?”	882 adults living with family members Women: 53% White: 79% Married: 70% Children in household: 43% Many years of education: 15% Employed full time: 47%	53%: family meals seven or more times per week 8%: eat one or two family meals per week 7%: never eat together ➢ Difficult to define a “typical week”

**Table 4:** Key findings from qualitative studies of family meals.

Key Results	Example of Empirical Evidence		References
The practices of family meals are socially situated	Conversations	Middle classes: emphasis on family mealtime conversations and particularly with children	De Vault 1991 (US) Morgensten et al. 2015, (France)
		Working class: conversations seem less important	De Vault 1991 (US)
	Negotiation of food choices	Higher classes: important that all family members eat the same food during the meals, leaving less room for negotiations with children (control over children’s diet)	Maurice 2015 (France) Wright et al. 2015 (Australia)
		Lower classes: children have more agency in the choice of the food they eat	Maurice 2015 (France) Wills et al. 2008 (Scotland)
	Conviviality	Middle classes: meals are expected to be a convivial moment conviviality as social distinction	Phull et al. 2015 (France)
Barriers to having regular family meals	Scheduling conflicts: school, extracurricular activities and adult work		Middleton et al. 2019 (international review)Jarrett 2016 (US) Malhotra 2013 (US) Bowen et al. 2019 (US) Martinasek et al. 2010 (US) Berge et al. 2013 (US) Trofholz et al. 2018 (US) Backett-Millburn et al. 2010 (Scotland) Gallegos et al. 2011
	Lack of time because of household chores that are done while children eat		
	Scarcity of help for the meal preparation		
	Limited resources (money and space to have family meals)		
	Parent(s) being too tired to eat with the children		
	Lack of ideas or confidence		
	Children characterised by parents as “picky eater”		
	Other activities are prioritized over family meals (sports, etc.)		
Challenges during family meals	Children’s physical behaviour characterised as “disruptive” by parents (i.e., not sitting “properly”, being “messy”, “improper” use of utensils)		Wilk 2010 (US) Malhotra 2013 (US) Berge et al. 2018, US, Trofholz et al. 2018 (US) DeVault 1991 (US) Berg et al. 2018 (US)
	Children characterised by parents as “picky eaters”, food refusal (also linked to resistance of parental authority)		
	Children’s behaviours characterised as difficult by parents: fighting or playing between sibling		
	Improper discussion or not enough discussion		
	Mealtime synchronisation: family member eating too quickly or too slowly		
	Family members being tired and strategic efforts to prevent usual conflicts become difficult		
Family mealtimes are gendered events	Middle class women: emphasis on conversations with children during meals and some women from working class also strive to construct the meal as family communication occasion, which constituted source of conflict with husband		De Vault 1991, US
	Link between mothers’ domestic food role with family cohesion and conviviality		Phull et al. 2015 Fournier et al. 2015 Kinser 2017

## Data Availability

Not applicable.
